# Recurrent parvovirus B19 viremia resulting in two episodes of hemophagocytic lymphohistiocytosis

**DOI:** 10.1186/s12985-022-01841-y

**Published:** 2022-06-25

**Authors:** Hans Martin Orth, Andre Fuchs, Nadine Lübke, Björn-Erik Ole Jensen, Tom Luedde

**Affiliations:** 1 Department of Gastroenterology, Hepatology and Infectious Diseases, Medical Faculty and University Hospital Düsseldorf, Heinrich-Heine-University, Düsseldorf, Germany; 2 Internal Medicine III - Gastroenterology and Infectious Diseases, Augsburg University Hospital, Augsburg, Germany; 3 Department of Virology, Medical Faculty and University Hospital Düsseldorf, Heinrich-Heine-University, Düsseldorf, Germany

**Keywords:** Macrophage activation syndrome, HLH, HScore, Relapse

## Abstract

**Background:**

Hemophagocytic lymphohistiocytosis (HLH) is a hyperinflammatory condition with uncontrolled activation of lymphocytes and macrophages. Besides a primary (genetic) form, HLH can also be triggered by malignant, autoimmune and infectious diseases. HLH recurrences are rarely described, usually only in primary HLH. *Parvovirus B19* (PVB19) Infection is one of the rare and rather benign causes of HLH. Since the infection usually results in long-lasting immunity, recurrent viremia is very uncommon.

**Case presentation:**

We report an unusual case of a young female with recurrent PVB19 infection that led to repeated episodes of HLH. The first episode occurred at the age of 25 years with a three-week history of high fever and nonspecific accompanying symptoms. The diagnosis of HLH was confirmed by HLH-2004 criteria and HScore, PVB19 viremia was detected as underlying cause. Following guideline-based therapy, the patient was symptom-free for one year, before similar symptoms recurred in a milder form. Again, PVB19 was detected and HLH was diagnosed according to HScore. After successful treatment and a nine-month symptom-free interval, a third phase of hyperinflammation with low PVB19 viremia occurred; this time, treatment with a corticosteroid and intravenous immunoglobulin was initiated before the presence of clear diagnostic criteria for HLH. No further events occurred in the following three years.

**Conclusions:**

In the case of our patient, the recurrent viremia triggered three episodes of hyperinflammation, two of which were clearly diagnosed as HLH. To our knowledge, this is the first published case of recurrent HLH due to PVB19 infection. Therefore, the case gives new insights in triggering mechanisms for HLH.

## Background

Since its first description in 1939 [1], hemophagocytic lymphohistiocytosis (HLH) has been subclassified into various entities including primary (genetic) and various secondary forms. Common to all entities is a hyperinflammatory state with uncontrolled activation of lymphocytes and macrophages, often—but not necessarily—resulting in manifest hemophagocytosis and critical illness [2]. Diagnostic criteria for the primary form have last been updated in 2007. Hemophagocytic lymphohistiocytosis is confirmed if at least five out of eight diagnostic criteria are met. These criteria comprise cytopenia, hypertriglyceridemia and/or hypofibrinogenemia, hyperferritinemia, elevated soluble CD25 (each above defined levels), and fever, splenomegaly, hemophagocytosis, and impaired NK cell activity [3]. The HLH-2004 criteria were generally used for secondary HLH (sHLH) as well; only in 2014, with the HScore, diagnostic criteria for secondary forms of HLH have been introduced. HScore is calculated as a sum of nine variables corresponding to different clinical and laboratory characteristics including immunosuppression, fever, hepatosplenomegaly, cytopenia, hyperferritinemia, hypertriglyceridemia, hypofibrinogenemia, AST elevation and hemophagocytosis. The sum values are associated with different probabilities of a correct diagnosis of HLH, the optimal cut-off is 169 [4].

Infection associated sHLH has first been described in 1979 as a hyperinflammatory condition following viral infections [5]. Since then, different pathogens have been described as triggers for sHLH, including viruses, bacteria, fungi and protozoa. The most frequent causative agent, however, appears to be *Epstein-Barr-Virus* [6]. *Parvovirus B-19* (PVB19) has rarely been identified as causative agent for sHLH, with a usually rather benign course of disease. Several cases of PVB19-induced sHLH were associated with hematologic disorders [7].

Infection with PVB19 usually results in sustained immunity against the virus. Persisting infection and recurrences have rarely been reported in immunocompetent individuals [8, 9]. In the following, we describe the case of an otherwise healthy female, who suffered repeated episodes of HLH during episodes of PVB19 viremia within a period of two years.

## Case presentation

Our patient’s first episode of sHLH occurred at the age of 25 years. She was admitted to hospital because of high fever, cough, joint pain, and dizziness that had been present for three weeks. She did not report any pre-existing conditions or a family history of autoimmune diseases. Physical examination was unremarkable. Diagnostic work-up revealed an active PVB19 infection with a positive PCR with 1,761 IU/ml and high level of IgG (Index > 46, reference < 0.9) but no detection of IgM antibodies (LIAISON® Biotrin Parvovirus B19 IgG; IgM). Further diagnostic testing revealed no evidence of underlying immunodeficiency or concomitant malignant, infectious or autoimmune disease (including CT scan of neck, chest and abdomen, abdominal and cardiac ultrasound, interferon gamma release assay for tuberculosis, serologies for HIV, CMV, EBV, viral hepatitis A, B, C, E, *Treponema*, *Bartonella*, *Brucella*, *Coxiella*, *Legionella*, *Leptospira*, *Leishmania*, and *Toxoplasma*, as well as ANA, ENA, ANCA, anti-LKM-1, AMA-M2, ASMA, anti-CCP and rheumatoid factors).

Tricytopenia, pronounced elevation of ferritin, splenomegaly, and elevation of soluble CD25 confirmed the suspected diagnosis of sHLH. A bone marrow biopsy was performed, but the material obtained was of low quality and therefore diagnostically inconclusive with regard to possible hemophagocytosis or detection of PVB19-DNA. Under therapy with corticosteroids, etoposide (four doses of initially 50 to finally 100 mg/m^2^) and intravenous immunoglobulins (IVIG) according to the current treatment guideline [10], disease remission of HLH and control of PVB19 infection were achieved. One year later, similar symptoms recurred. With evidence of PVB19 viremia (275 IU/ml) and elevation of ferritin and tricytopenia, the clinical diagnosis of sHLH relapse and recurrent replication of PVB19 was made and successfully treated with corticosteroids and intravenous immune globulin (IVIG). A third episode of HLH occurred another nine months later with detection of a low amount of PVB19-DNA (2 IU/ml) and elevated inflammatory biomarkers. Although the diagnostic criteria for HLH were not fully met, we considered the comparatively mild symptoms to be incipient sHLH and started treatment with IVIG and prednisolone, which resulted in rapid resolution of symptoms. The patient has since been healthy without relapses for three years. Table 1 gives details of clinical and diagnostic characteristics of the three episodes.

**Table 1:** Diagnostic and clinical characteristics of the three episodes and follow-up appointments

Parameter	Unit	reference values	1^st^ episode	follow-up	2^nd^ episode	follow-up	3^rd^ episode
			(t0)	2 months	12 months	15 months	21 months
CRP	mg/dl	< 0.5	**12.2**	< 0.1	**1.3**	< 0.1	**1.5**
PCT	ng/ml	< 0.05	**10.38**	..	**0.18**	..	..
LDH	U/l	< 247	**2844**	215	**653**	..	337
GOT (AST)	U/l	< 31	**2635**	17	**105**	15	31
Ferritin	µg/l	13,0–150,0	**24,068**^**#**^	**179.4**	**9864**^**#**^	65.2	**1323**^**#**^
sCD25 (sIL-2R)	kU/l	158–623	**3822**^**#**^	..	**1136**	..	490
White blood count	nl^−1^	4–11	**0.6**	4.4	**2.6**	10.7	5.8
Neutrophiles	nl^−1^	2.0–8.3	**0.3**^**#**^	..	**1.2**	..	4.22
Platelets	nl^−1^	150–400	**48**^**#**^	212	**66**^**#**^	247	179
Hemoglobine	g/dl	11.9–14.6	**6.3**^**#**^	13.4	11.0	14.4	10.5
Fibrinogen	mg/dl	210–400	236	..	254	..	**401**
Triglycerides	mg/dl	< 150	**229**	54	**140**	..	..
NK cell activity		..	..	..	..	..	Normal
Parvovirus B19-DNA	IU/ml	Negative	**1761**	Negative	**275**	..	**2**
Temperature	°C		**40.4**^**#**^		**39.0**^**#**^		36.6
Spleen length	mm	< 110	**161**^**#**^	**112**	**124**^**#**^		109
Liver size (cc and dv in MCL)	mm		**( +) 110/167**	112/144	122/128		131/140
Hemophagocytosis	Yes/no	No	(−)*		..		..
HLH2004	n	< 5	**5**		3		1
Hscore	n		**264**		**193**		19
Corresp. likelihood	%		** > 99**		**80–88**		< 1

CRP: c reactive protein; PCT: procalcitonin; LDH: lactate dehydrogenase; sCD25 (sIL-2R): soluble CD25 (soluble interleukin-2 receptor); WBC: white blood count; cc: cranio-caudal; dv: dorso-ventral; MCL: mid-clavicular line; ..: no data/not conducted. *: aspirate of low quality and therefore not diagnostically conclusive. Values in bold: pathologic findings (values beyond laboratory reference ranges). ^#^: meeting corresponding HLH-2004 criteria

After the third episode, we performed an additional immunologic workup: Immunophenotyping with fluorescence-based flow cytometry showed T-lymphopenia with only 219 CD4+ cells/µl despite repeated negativity for HIV. CD4+ and CD8+ cells showed normal HLA-DR expression. Additionally, few class-switched memory B-cells (IgD−/CD27+) were detected (3/µl), but total immunoglobulins including IgG-subclasses were within normal range. Absolute number of NK cells (CD56+/CD3−) was within normal range, NK cells showed normal perforin expression (intracellular staining with perforin antibody) and normal degranulation activity (CD107a) following stimulation with target cells compared to healthy controls. Genetic analysis of possible underlying causes of HLH was recommended to the patient following discharge, but ultimately not conducted.

## Conclusions

Reactivation or persistence of PVB19 viremia is an uncommon event in immunocompetent individuals. Interestingly, at the time of diagnosis of the first episode of HLH in our patient, high PVB19-specific IgG levels but no IgM were detected, suggesting that this episode was in fact already a reactivation of persistent PVB19 infection rather than a primary infection. In previous reports, the possibility of persistent infection of the bone marrow with PVB19 is discussed [7], unfortunately in our case, bone marrow biopsy did not yield adequate material for PCR. The PCR results from blood during the different hospital admissions show decreasing viral load over the three episodes, probably due to improved specific immunity by a CD4+ cell-mediated booster effect as known from other viral infections [11]. However, this could not be quantified, because repeatedly high concentrations of PVB19-specific IgG were measured (i.e.: index > 46) throughout the two-year course. The decreasing viremia went along with ameliorated HLH symptoms from first to third episode. Previous reports suggest that PVB19 induces rather benign courses of sHLH, which in some cases do not even require specific HLH therapy [6]. In other infectious triggers like malaria, spontaneous resolution of HLH symptoms has been shown with clearance of the infectious agent [12].

During the first episode, both HLH-2004 criteria and HScore unambiguously supported the diagnosis of sHLH. During the second episode, only three HLH-2004 criteria were met, while an HScore of 193 (corresponding to a calculated likelihood of 80–88%) conclusively indicated HLH. The detected PVB19 viremia was lower this time and symptoms were overall milder. We assume that this is due to the earlier diagnosis, since the treating physicians and the patient were aware of her medical history. Because of the less severe symptoms, no etoposide was administered. The diagnosis of a third episode of sHLH is ultimately elusive, as neither the HLH-2004 criteria nor the HScore provide clear evidence of HLH. Nevertheless, inflammatory biomarkers were elevated and PVB19 detectable. Considering the patient's medical history, we therefore made a diagnosis of incipient sHLH and initiated early treatment with prednisolone and IVIG, which quickly led to resolution of symptoms.

Regarding the diagnostic criteria, it should be mentioned that bone marrow aspiration during the first episode was not diagnostically meaningful due to low quality of the aspirate. Since the diagnosis was already confirmed, we decided against repeated bone marrow biopsy and against bone marrow puncture during the relapses. The absence of this value may have reduced diagnostic accuracy of HScore and HLH-2004 criteria during the second and third episode. Since bone marrow is a potential reservoir for PVB19, a bone marrow analysis for PVB19-DNA would also be helpful in assessing the course of infection and should therefore be sought in case of further relapse.

Hemophagocytic lymphohistiocytosis is usually considered a threshold disease, which—once a tipping point is exceeded—leads to uncontrolled, self-sustained hyperinflammation and macrophage activation [2]. Relapse of HLH has been described, but it is usually associated with primary HLH, which is caused by genetic disorders of CD8+ T cells and NK cells [13]. Although immune cell phenotyping in our case yielded no evidence of primary HLH, a genetic overlap between primary and secondary HLH is discussed [2]. Our patient’s case therefore provides new insights into possible long time prognosis of sHLH and suggests that the recurrence of triggering effects—in this case PVB19 viremia—is capable of inducing relapse of sHLH.

## Data Availability

All clinically relevant data have been made available within the manuscript. For data protection reasons, no further clinical data can be made available.

## Abbreviations

HLH: Hemophagocytic lymphohistiocytosis
PVB19: *Parvovirus B19*
sHLH: Secondary HLH
IVIG: Intravenous immune globulin
